# Metabarcoding reveals the dietary diversity and food web structure of spider functional guilds in a highly diverse subtropical forest

**DOI:** 10.1007/s00442-026-05956-9

**Published:** 2026-08-07

**Authors:** Jingting Chen, Mingqiang Wang, Arong Luo, Zhe Zhao, Feng Zhang, Douglas Chesters, Xiaoyu Shi, Andreas Schuldt, Chaodong Zhu

**Affiliations:** 1 CAS State Key Laboratory of Animal Biodiversity Conservation and Integrated Pest Management (SKLA2501), Institute of Zoology, Chinese Academy of Sciences, Beichen West Road 1, Beijing, 100101 China; 2 Key Laboratory of Mountain Ecological Restoration and Bioresource Utilization & Ecological Restoration Biodiversity Conservation Key Laboratory of Sichuan Province, Chengdu Institute of Biology, Chinese Academy of Sciences, Chengdu, China; 3 Department of Entomology, College of Plant Protection, Nanjing Agricultural University, Nanjing, 210095 China; 4 Forest Nature Conservation, University of Göttingen, Büsgenweg 3, 37077 Göttingen, Germany; 5 College of Biological Sciences, University of Chinese Academy of Sciences, No. 19A Yuquan Road, Beijing, 100049 China

**Keywords:** Araneae, Diet, Hunting mode, Metabarcoding, Network

## Abstract

Predator-prey interactions are key to regulating lower trophic levels and stabilizing ecosystem processes. Therefore, understanding prey selection and prey composition of predators is essential, yet especially the small size and diverse diets of predatory arthropods present a significant challenge to conventional field methods. Here, we employed DNA metabarcoding on gut contents of over 1500 arboreal spiders in a subtropical forest in China to construct a high-resolution spider-prey interaction network. Subsequently, we compared prey diversity, composition, starvation rates, and network metrics (predator niche overlap, generality, and prey vulnerability) across different spider hunting guilds and families. Our results revealed an exceptionally broad diet for these spiders, with spiders themselves constituting a significant proportion of prey (i.e. spider on spider predation), besides a wide range of insects, particularly flies and moths. Although, prey composition was broadly similar at high taxonomic levels and functional groups, distinct dietary partitioning was evident at lower (MOTU) taxonomic level. Interaction networks revealed a broader and more generalized diet spectrum of active-hunters compared to web-builders, which resulted in higher diet overlap within the hunting guild (high prey vulnerability and niche overlap). However, network metrics for active hunters were lower than null expectations, suggesting a structuring role of intraspecific competition, while web-builders displayed greater random associations, possibly reflective of the passive mode of prey capture. Collectively, our study offers a high-resolution overview of the dietary niches of subtropical arboreal spiders, revealing how hunting mode shapes their ecological impact and providing significant implications for leveraging these predators in biological control strategies.

**Supplementary Information:**

The online version contains supplementary material available at https://doi.org/10.1007/s00442-026-05956-9.

## Introduction

Predator-prey interactions are primary determinants of top-down effects in ecological systems (Finke and Denno 2004; Schmitz 2007), significantly influencing ecosystem functions such as nutrient cycling (Ngai and Srivastava 2006; Schmitz et al. 2010), primary productivity and pest control (Cain 1978; Perdikis et al. 2011). Understanding predator feeding ecology is therefore essential for evaluating their functions in ecosystems, including population regulation mechanisms and competitive interactions among co-occurring predators (Pringle et al. 2019; Wollrab et al. 2013).

Spiders represent the most ubiquitous generalist arthropod predators in terrestrial ecosystems (Greenstone 1999), estimated to consume in the realm of 400–800 million tons of prey each year (Nyffeler and Birkhofer 2017). As such, spiders play a major role in pest control, and have often been used successfully in this role in agriculture (Michalko et al. 2019). However, the biological control efficiency can be modulated by interspecific interactions, such as intraguild predation (spiders often feed on other spiders), which might reduce overall control efficiency (Finke and Denno 2006; Michalko et al. 2019). Additionally, spider diet variability can arise because spider dietary preferences are strongly influenced by their functional traits, such as hunting strategy and body size (Chapman et al. 2013; Perkins et al. 2018; Schmidt et al. 2012). For instance, sit-and-wait spiders specialize in capturing mobile prey, whereas actively hunting spiders are more effective at capturing sedentary prey (Sweeney et al. 2013). Further, while spiders are generally considered effective predators, they have been observed to experience starvation in the field, with web-building spiders showing greater starvation resistance than hunting spiders (Anderson 1974; Overgaard and Wang 2012). Previous research using stable isotope analysis and direct observation of prey remains in spider webs has provided insights into the diet composition and trophic niches of spiders, particularly web-building spiders (Kennedy et al. 2019; Michalko et al. 2021a; Zuev et al. 2020). However, significant knowledge gaps remain regarding the high-resolution trophic dynamics of diverse spider communities in complex forest ecosystems. More detailed dietary analysis is required to obtain insights into spider niche partitioning and to better resolve the trophic structure of predator-prey interactions in these ecosystems.

Dietary analysis is a fundamental means to define interactions networks at various trophic levels. Predators might vary in prey range (specialization versus generalization), display resource partitioning or overlap in their prey species (niche overlap), while a given species may be preyed upon by a variable number of predator species (prey vulnerability). However, characterizing predator-prey networks is methodologically challenging due to small body size, high mobility, habitat diversity, and dietary complexity of the species involved, particularly for generalists. Traditional dietary analysis methods include field observations, laboratory rearing, analysis of prey remains on webs, and stable isotope analysis. However, these approaches demand taxonomic expertise, are laborious (Kaunisto et al. 2017), and their accuracy can be limited in some cases, such as those involving external digestion and high mobility (Furlong 2015). In order to address some of these shortcomings, molecular techniques have been increasingly applied both to gut contents and fecal samples (Sheppard and Harwood 2005). Particularly, DNA metabarcoding has emerged as a powerful tool for identifying diverse prey taxa (Coissac et al. 2012; Ji et al. 2013; Taberlet et al. 2012). Compared to traditional approaches, high-throughput sequencing offers superior sensitivity for detecting trace DNA while enabling species identification across taxa without requiring morphological expertise. For small predatory arthropods, this approach coupled with short-fragment primers effectively recovers highly degraded prey DNA from digestive systems (Zeale et al. 2011). However, these molecular approaches are still constrained by their inability to enumerate individual prey items, thereby hindering precise quantification of energy flux or the top-down impacts on prey population dynamics. Nevertheless, DNA metabarcoding remains an indispensable tool for elucidating complex multi-trophic linkages, which constitutes the primary objective of the present study.

To disentangle the network structure and ecosystem function difference of spider guilds, we employed DNA metabarcoding to analyze the dietary composition of arboreal spiders in a subtropical forest. Spiders were classified by hunting mode (web-building and actively hunting) and subdivided into dominant families (Cardoso et al. 2011). We evaluated prey richness, starvation ratio, and predator-prey network indices (predator generality, niche overlap, and prey vulnerability) to compare dietary differences among spider groups. The starvation rate, calculated as the percentage of individuals with no detectable prey DNA, was used to represent the frequency of empty guts. This allowed us to assess the immediate foraging success and the top-down predation pressure at the time of sampling. First, we hypothesized that hunting and web-building spiders exhibit distinct dietary patterns and network indices (Birkhofer et al. 2022; Birkhofer and Wolters 2012). Specifically, we predicted that (a) active hunting spiders, characterized by high mobility and aggression, have a broader prey spectrum (higher generality) and a lower starvation rate, and concurrently show stronger niche differentiation (i.e. lower niche overlap) to reduce competition with other active hunters. In contrast, web-building spiders, employing a sit-and-wait hunting strategy, face less stable food resource and require greater starvation resistance. Consequently, we expected them to have a narrower and compositionally more similar prey spectrum, leading to a higher potential for niche overlap. Second, we hypothesized that dietary richness and composition is driven by family-specific foraging traits. Among web-building spiders, we posited that (b) family constructing structurally complex 3D webs (e.g. Theridiidae) capture a greater diversity of prey compared to those utilizing 2D orb webs (e.g. Araneidae). Among hunting spiders, we predicted that (c) the highly mobile and aggressive families (e.g. Oxyopidae and Salticidae) exhibit a wider prey spectrum than ambush predators (e.g. Thomisidae) which are expected to have a more specialized diet.

## Materials and methods

### Study site and sampling

The BEF-China experiment, planted in 2009/2010 and located in Jiangxi province, China (29°08′-29°11′N, 117°90′-117°93′E), is the largest tree diversity experiment worldwide (Bruelheide et al. 2014). The climate is typical subtropical, with a mean annual temperature of 16.7℃ and mean annual precipitation of 1,821 mm (Yang et al. 2013). The temperature ranges from 0.4℃ to 34.2℃ from the coldest to the warmest month of the year (Yang et al. 2013). The species pool at this study site comprises 40 evergreen tree species planted in mixtures with up to 24 tree species. The natural forest in this region consists of a mixture of evergreen and deciduous species. To obtain a comprehensive sample of arboreal spiders, we sampled a total of 4,240 trees using branch beating and shaking methods from a total of 53 plots that reflected and allowed us to generalize across the experimental design of BEF-China (Chen et al. 2023; Wang et al. 2019). Within each plot, where 400 trees were planted in a 20 × 20 grid, we sampled 80 individual trees. Spiders that fell on the beating sheets (1.5 × 1.5 m) were collected and individually preserved in vials containing 99.9% ethanol. All specimens were subsequently stored at -20 °C for further treatments. This study exclusively involved invertebrate (Arachnid) specimens, which do not require special permits or animal care approvals for field collection or laboratory analysis.

### Spider identification

Spiders were classified into morphospecies (Derraik et al. 2002). To mitigate potential bias arising from sexual dimorphism, we employed a conservative protocol in which morphologically distinct individuals were initially categorized as discrete morphospecies to prevent premature lumping. Subsequently, DNA barcoding (targeting the mitochondrial cytochrome c oxidase subunit I gene, COI) was performed to validate and reconcile these taxonomic delineations. For each morphospecies, three randomly selected individuals were sequenced. If all three individuals were assigned to the same Molecular Operational Taxonomic Unit (MOTU), the morphospecies was confirmed. Crucially, if barcoding revealed that different morphospecies shared the same MOTU (e.g., bridging morphologically distinct males and females), they were merged into a single species-level taxon. In cases where sequences within a morphospecies were inconsistent, all individuals in that group were sequenced to ensure accurate delineation. Additionally, adult spiders were selected for inclusion in a local reference database that also included morphological and DNA barcode identification. Morphological identification of adults was performed by Professor Jie Liu at Hubei University. 1–2 legs of each spider were used for DNA extraction, following the standard process of TIANamp Genomic DNA Kit (DP304-03). PCR was prepared with 30ul reaction volumes, containing 15 µL Premix PrimeSTAR HS (TaKaRa), 10 µL ddH2O, 1 µL forward/reverse primers (LCO1490 / HCO2198), and 3 µL template DNA. PCR conditions were 94℃ for 4 min, followed by 35 cycles at 94℃ for 45 s, 45℃ for 40 s, 72℃ for 2 min, and a final extension at 72℃ for 10 min. PCR products were examined on 1% agarose gel. PCR products with strong, single bands were sequenced using BigDye v3.1 on an ABI 3730xl DNA Analyser (Applied Biosystems).

The raw sequences were first aligned using MAFFT v7.0 (Katoh et al. 2002), and subsequently edited in BioEdit v7.0.5. They were then translated into amino acid sequences using MEGA X (Kumar et al. 2018) to check for the presence of stop codons. The ‘unique.seqs’ command of MOTHUR (Schloss et al. 2009) was used to dereplicate the sequences into haplotypes. The haplotype sequences were clustered into MOTUs using five species delimitation methods, including the BLASTclust module in the NCBI-BLAST package (97.8% similarity; Ratnasingham and Hebert 2013), ABGD (settings: Kimura (K80) model, Pmin = 0.001, Pmax = 0.1, Steps = 50, X = 0.5, and Nb bins = 50; Puillandre et al. 2012), bPTP (based on the maximum likelihood phylogeny), Mothur (furthest neighbor algorithm), and jMOTU (97% similarity cutoff). The degree of agreement among the five methods was evaluated using the Hubert and Arabie-adjusted Rand index with the CLUES package (Wang et al. 2007), and the most consistent results were used for subsequent analysis (Wang et al. 2019). Spider MOTUs were identified using Statistical Assignment Package (SAP; Munch et al. 2008a, b), with a reference database comprising the local spider COI barcode database and the BOLD online database (The Barcode of Life Data System; Ratnasingham and Hebert 2007).

### Prey detection

To detect residual prey DNA in spider guts, we performed metabarcoding analysis on the entire spider abdomen, which retains higher concentrations of prey DNA compared to other body parts (Krehenwinkel et al. 2016; Macías-Hernández et al. 2018). Spiders were rinsed in ethanol to reduce potential external contaminants. We measured spider dry weight and then cut the spider abdomen for further use. Each sample was individually ground after being frozen with liquid nitrogen, and genomic DNA was extracted following the standard protocol of the Qiagen DNeasy Tissue kit (Qiagen). DNA was amplified in a single PCR step for the DNA barcode region using primers ZBJ-ArtF1c and ZBJ-ArtR2c (Zeale et al. 2011), producing a 157 bp amplicon (Stothut et al. 2024). Forty pairs of primers were labeled at their 5’ end with a unique 8 bp barcode, allowing for the assignment of sequences to their original samples (e.g. F1-R1, F2-R2) during bioinformatics analysis. PCRs were carried out in 25 µL reaction volumes, containing 18 µL of Premix PrimeSTAR HS (TaKaRa), 3 µL of ddH2O, 1 µL of forward/reverse primers, and 2 µL of template DNA. Thermocycler conditions were as follows: initial denaturation at 95℃ for 3 min; 30 cycles of 30 s at 94℃, 30 s at 50℃, 2 min at 65℃, and a final extension for 5 min at 65℃. Each sample had three PCR replications. Amplicons were pooled and purified using the PCR purification kit (Vazyme). The purified PCR products were quantified using a Qubit fluorometer, and an equal amount of DNA from each PCR product was pooled. Sequencing was carried out on an Illumina NovaSeq 6000 platform (2 × 150 bp paired-end reads).

The bioinformatics analysis of spider gut content followed a standardized pipeline using fastp (Chen et al. 2018), CUTADAPT v4.2 (Martin 2011) and DADA2 (Callahan et al. 2016). Raw sequencing reads were first processed for quality control using fastp. Paired-end reads were demultiplexed based on exact barcode matching (single mismatches), followed by subsequent trimming of primers and barcodes using CUTADAPT v4.2. Then, the DADA2 pipeline was employed to perform quality filtering, sample inference, chimera identification, and merging of paired-end reads, culminating in the generation of an Amplicon Sequence Variants (ASVs). All DADA2 steps were performed in R v4.4.2 (http://www.R-project.org). To identify and remove host DNA contamination, the most abundant sequence in each gut content sample was provisionally designated as the host spider sequence, which was cross-validated against sequences obtained from corresponding spider leg tissue. Sequences exhibiting greater than 93% similarity to the host spider sequence were subsequently filtered out to minimize potential host contamination. The intraspecific and interspecific genetic distance thresholds for the spiders in this study were determined based on the calculated pairwise genetic distances among host spider sequences using the K80 substitution model (Figure S1). This data-driven approach ensured that host-specific variants and potential sequencing errors were effectively removed without inadvertently discarding closely related spider prey sequences. Remaining sequences were clustered into MOTUs based on a 97% similarity threshold, which is the most widely adopted consensus for species-level delineation in arthropod dietary studies using these specific COI mini-barcodes (e.g., Jusino et al. 2019; Penner et al. 2024). Taxonomic classification was performed using SAP against a local arthropod database downloaded from the BOLD system. Taxonomic assignments were further validated by conducting a search against the NCBI nucleotide collection (nr/nt) database using BLASTn (Basic Local Alignment Search Tool; Sayers et al. 2022). For each MOTU, the top BLAST hit (lowest E-value) was designated as the final taxonomic classification. To minimize potential false positives and rare artifacts, prey MOTUs were filtered: MOTUs containing fewer than 5 reads and concurrently exhibiting a relative abundance below 0.001 were removed from the final dataset. Prey MOTUs were subsequently assigned to functional feeding guilds (herbivores, carnivores, decomposers, and others) based primarily on family-level taxonomic assignments.

### Statistical analysis

All statistical analyses were conducted in R v.4.4.2 (http://www.R-project.org) using the packages vegan (Oksanen et al. 2019), bipartite (Dormann et al. 2008) and iNEXT (Hsieh et al. 2016). The analyses were performed for the overall dataset based on all spider specimens, as well as separately for the two hunting modes (web-building and active hunting) and the five dominant spider families (Araneidae, Theridiidae, Oxyopidae, Salticidae, Thomisidae). We calculated spider starvation rate, prey richness and composition, and spider-prey network indices (generality, vulnerability and niche overlap). We defined the starvation rate as the proportion of spider individuals with no prey detected. Given that the average half-life for molecular prey detection in spiders is estimated to be approximately 83 h (Macías-Hernández et al. 2018; Uiterwaal and DeLong 2020), this metric provides a snapshot of the foraging success and trophic pressure within the spider community. Generality describes the number of prey species consumed by a spider species, while vulnerability describes the number of spider species consuming a prey species. Niche overlap quantifies dietary similarity among predator species. It is currently not possible to estimate the absolute abundance of prey using metabarcoding, due to inherent biases related to prey body size, tissue digestibility, and differential DNA recovery rates (Pompanon et al. 2012). To avoid these quantitative biases, we utilized occurrence-based metrics (presence/absence of prey DNA) for all dietary analyses. We summarized and compared the prey richness and composition for all spiders, and then for the guilds separately (hunting vs. web-building, and the five dominant family guilds). We further summarized the prey of different spider guilds into functional groups (herbivores, carnivores, decomposers, and others) based on family. To standardize comparisons of prey richness among spider groups of varying diversity, we used the R package iNEXT to generate rarefaction and extrapolation curves from a binary matrix of prey MOTU presence/absence within each spider MOTU. Based on the relationship between spiders and their prey, we constructed interaction networks for different spider guilds. To assess whether the observed network indices deviated significantly from random expectations, we compared them against null models generated using the Patefield algorithm (10000 permutations), which maintains marginal totals. The differentiation in prey composition among spider hunting strategy and dominant family guilds was quantified using nonmetric multidimensional scaling (NMDS) analysis with Bray-Curtis distances. We selected two dimensions (k = 2) based on the reduction in stress value, and subsequently centered and rotated the results using principal components to maximize the variance explained by the first axis. To mitigate potential biases arising from uneven sample size across different spider guilds, we performed a standardized bootstrap resampling procedure (999 iterations). For each iteration, we equalized the sampling intensity by randomly resampling 100 individuals (with replacement) from each group. This unified framework was applied to both dietary richness and compositional analyses.

To verify the authenticity of the high intraguild predation observed, we quantified the frequency of spider-on-spider predation across all spider families and visualized the results with a heatmap. Moreover, we conducted a sensitivity analysis using a restricted dataset that included only insect prey.

## Results

A total of 1,558 spiders, representing 15 families, were collected from the field. The five most dominant families were Araneidae (*N* = 942), Salticidae (*N* = 228), Thomisidae (*N* = 151), Oxyopidae (*N* = 118), and Theridiidae (*N* = 48). These specimens were categorized by hunting guild into 1,017 web-building and 541 active hunting spiders. Among them, we detected prey DNA in 504 individuals (representing 81 spider MOTUs) after applying all the filters.

**Fig. 1:**
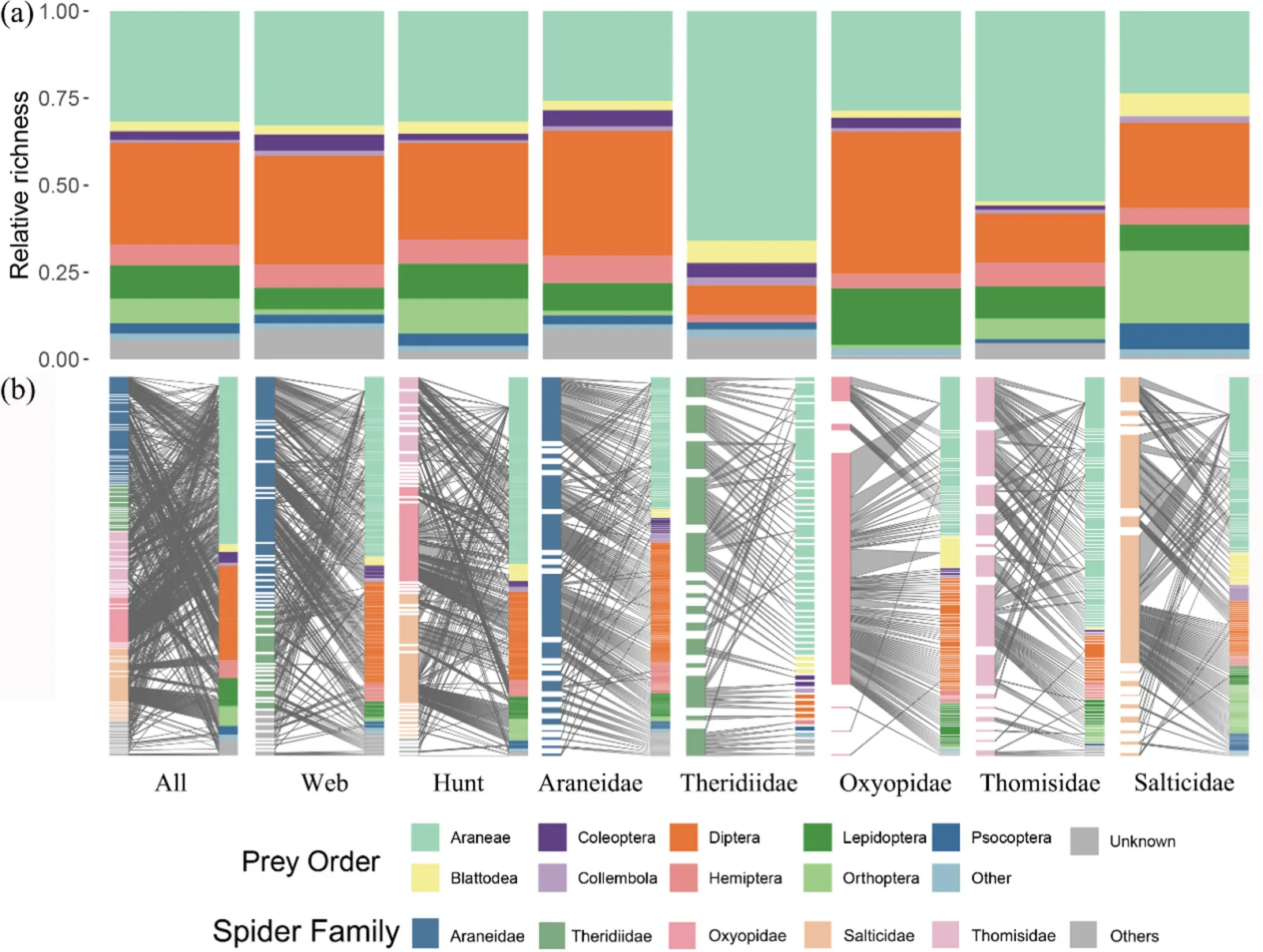
(**a**) The relative richness of prey detected in all spiders, spiders of two hunting modes and five dominant families. (**b**) Corresponding predator-prey networks resolved at MOTU level, where each box represents an individual MOTU and lines show feeding connections. The left column represents the predator spiders, and the right column represents the prey MOTUs

We identified a total of 397 prey MOTUs spanning 14 taxonomic orders. The diet was taxonomically dominated by Araneae (126 MOTUs, 31.70% of total richness), Diptera (116 MOTUs, 29.20%), and Lepidoptera (38 MOTUs, 9.57%), which together accounted for 70% of the prey richness (Fig. 1a; Table 1). Other common orders included Orthoptera, Hemiptera, and Blattodea. In terms of functional composition, the prey spectrum was dominated by carnivores (38.67%), followed closely by herbivores (33.07%) and decomposers (26.13%) (Fig. 4).

**Table 1:** Prey richness, starvation rate and network indices for all spiders, spiders in two hunting modes (web-building and hunting) and dominant families (Araneidae, Theridiidae, Oxyopidae, Salticidae, Thomisidae)

	Prey richness	Starvation rate	Niche overlap	Generality	Vulnerability
All	397	67.65	0.12*	19.98*	6.22*
Web	195	81.32	0.08	19.14	3.93
Hunt	259	41.96	0.16*	20.39*	4.21*
Araneidae	151	85.03	0.04	25.43*	1.88*
Theridiidae	47	35.42	0.12	7.10*	2.58
Oxyopidae	98	20.34	0.06*	31.75*	1.23*
Thomisidae	106	24.50	0.35	16.43*	2.32*
Salticidae	86	57.02	0.13*	12.84*	2.60*

Asterisks (*) indicate values that were significantly lower than expected from null model analyses

**Fig. 2:**
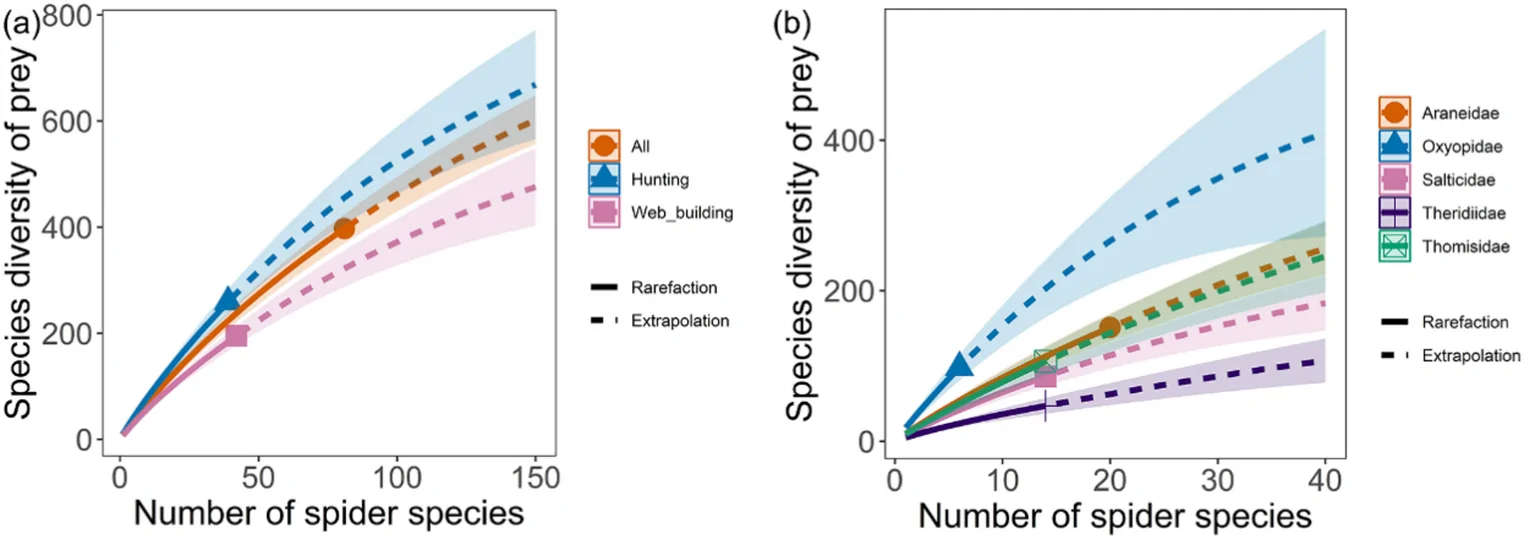
Rarefaction and extrapolation sampling curves of prey richness in relation to spider species richness. The curves illustrate the accumulation of prey species richness as a function of the number of spider species sampled for (**a**) the total dataset and the two hunting guilds, and (**b**) the five dominant spider families. Shaded areas represent 95% confidence intervals

Active hunting spiders exhibited a significantly higher estimated prey richness than web-building spiders when standardized to equal sample sizes (standardized *N* = 150, yielding 669 vs. 476 MOTUs; Fig. 2a). Furthermore, active hunting spiders displayed a significantly lower starvation rate (41.96% vs. 81.32%; Table 1). Regarding diet composition, intraguild predation (spider-on-spider predation) was the dominant feeding strategy across all spider guilds, followed by Diptera prey (Fig. 1a). While the two feeding guilds exhibited substantial overlap in their prey spectra at the taxonomic order level (Figure S2a) and functional group level (Fig. 4a), their specific prey spectra (MOTU level) differed (Fig. 2a). Consequently, niche overlap indices of spider-prey networks at MOTU level were low (Table 1). In terms of network structure, hunting spiders displayed higher niche overlap and slightly higher values of generality and vulnerability compared to web-building spiders (Table 1; Fig. 1b).

Prey richness and starvation rate varied substantially among the five dominant families (Table 1). Oxyopidae (410 MOTUs) displayed the highest estimated prey richness (standardized *N* = 40), followed by Araneidae (256 MOTUs), Salticidae (245 MOTUs), Thomisidae (183 MOTUs), and Theridiidae (107 MOTUs) (Fig. 2b). Conversely, Araneidae showed the highest starvation rate (85.03%), while Oxyopidae exhibited the lowest (20.34%) (Table 1). Network analysis revealed distinct trophic roles among the spider families: Oxyopidae and Araneidae displayed the highest generality, Salticidae and Theridiidae showed maximal vulnerability, while Thomisidae exhibited the greatest niche overlap (Table 1). The diets of Theridiidae and Salticidae were dominated by carnivores. In contrast, Araneidae and Thomisidae consumed a substantial proportion of herbivores, whereas Oxyopidae preyed primarily on decomposers (Fig. 4).

**Fig. 3:**
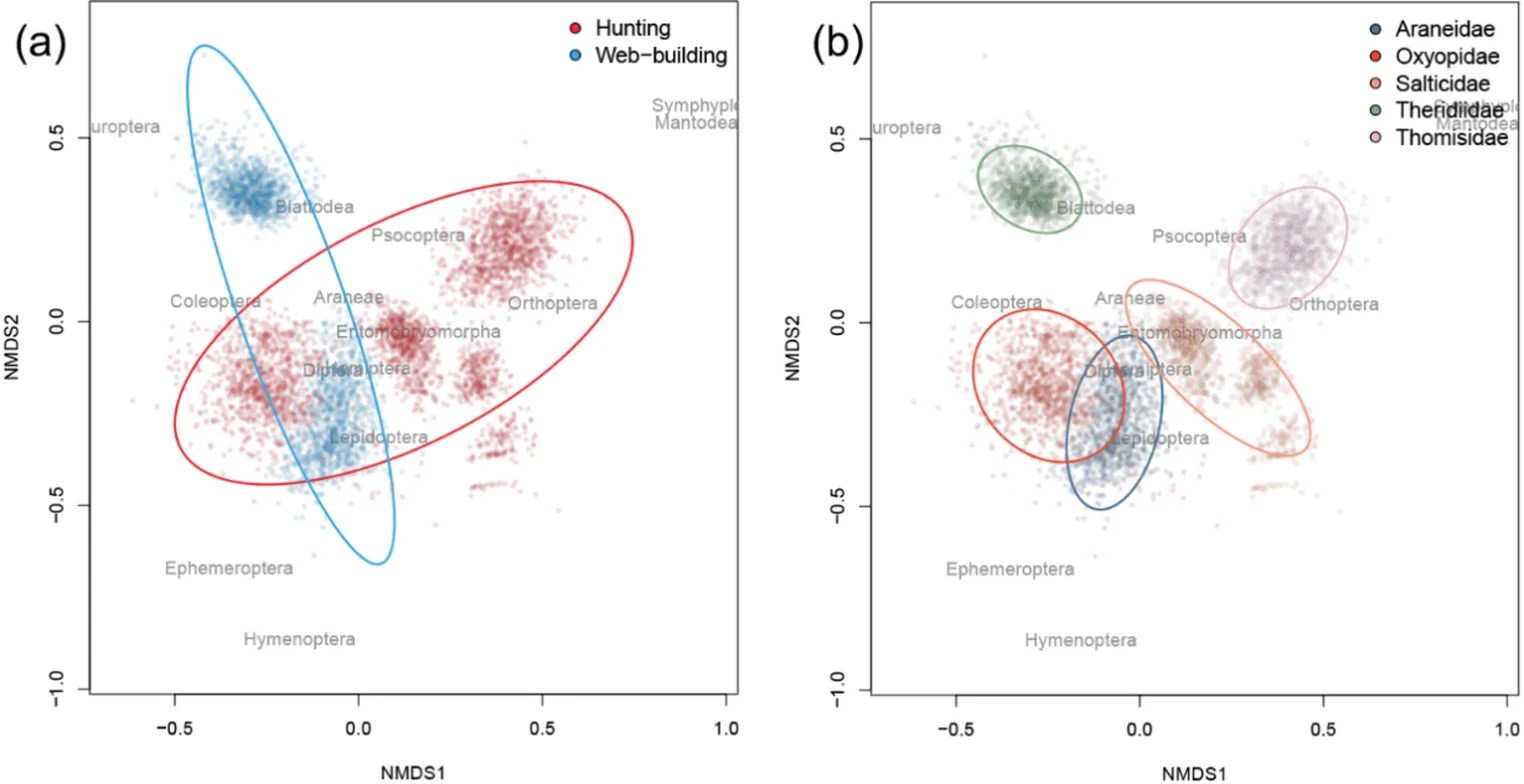
Non-metric multidimensional scaling (NMDS) ordination of spider dietary composition based on a unified bootstrap resampling framework (*n* = 100 individuals per group, 999 iterations). (**a**) dietary differentiation among the five dominant spider families; (**b**) dietary differentiation between hunting and web-building guilds

Based on a unified bootstrap resampling procedure, we found that the mean relative richness across guilds was highly consistent with the primary patterns (Figure S3). Notably, while the raw dietary data suggested substantial overlap at the taxonomic order level, the standardized bootstrap NMDS ordination revealed clearly separated dietary niches among the different guilds (Fig. 3). The observed values for the network indices of most guilds deviated significantly from random expectations. Exceptions were found for overall web-building spiders, the niche overlap indices of Araneidae, Theridiidae, and Thomisidae, and the vulnerability specifically of Theridiidae (Table S1).

**Fig. 4:**
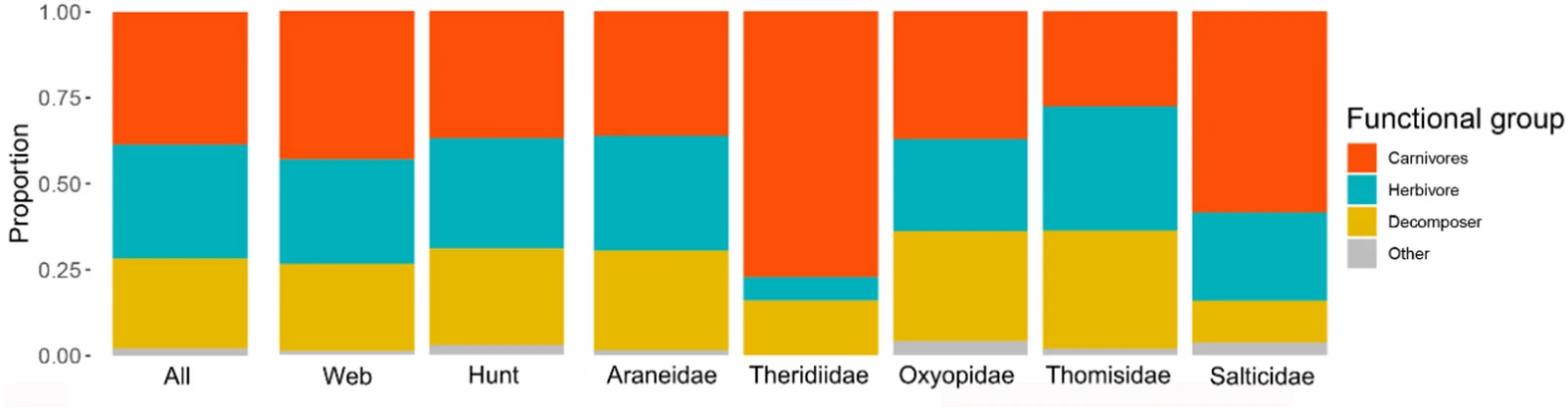
Proportion of prey in various functional groups captured by spiders (all, two hunting modes and five dominant families)

Based on the heatmap of spider-on-spider predation, Nephilidae was the most frequently detected prey family across most predator groups, particularly with the diet of Thomisidae and Oxyopidae. Across the broader community, while this pattern was consistent among the dominant taxa, it was not detected in every one of the 15 sampled families (Figure S4). Upon excluding Araneae prey, the community-level starvation ratio increased from 67.6% to 81.8% (Table S2). However, the relative ranking of predator generality among families remained consistent with the full dataset, with Oxyopidae maintaining the highest value (Generality = 47.3), followed by Thomisidae and Araneidae. Furthermore, niche overlap indices for all guilds and families decreased to lower absolute values, ranging between 0.01 and 0.03, compared to the primary analysis (Table S2). The overall structure of the predator-prey networks and the ordination patterns in the NMDS plots (Fig. S7) remained similar to those derived from the full dataset.

## Discussion

Employing large-scale metabarcoding, we investigated the diet composition and potential functional divergence of arboreal spiders within a subtropical forest. We found substantial intraguild predation during the sampling period, with insects a secondary food resource. When comparing the diet between spiders with different hunting strategies, we found that active hunters consumed a broader prey spectrum and showed a lower starvation rate than web-building spiders. Despite substantial dietary overlap, the two guilds demonstrated distinct taxon-specific foraging preferences in terms of prey proportions, although these differences further varied at the family level. Our metabarcoding analyses thus help to develop a deeper understanding of the still obscure food web structure of arthropod communities and the functional role of spiders in forest canopies.

We observed a remarkably high starvation rate among spiders at the study site. Adequate food is essential for meeting the energetic demands of growth and reproduction, which is a primary constraint on spider community fitness. This is particularly relevant for predators like spiders, as their prey availability often exhibits high spatio-temporal variability due to factors such as seasonality and environmental conditions (Wang et al. 2025). For example, habitat complexity, such as dense forest structures, can increase prey search time and reduce predation efficiency (van Schalkwyk et al. 2021). Moreover, among the spiders with detectable prey DNA, a high proportion of the diet consisted of intraguild prey (spider consuming other spiders), which is unexpected based on classic visual studies (Birkhofer et al. 2022). This high rate likely represents a genuine biological signal rather than a methodological artifact. This highlights the advantage of high-throughput-based molecular methods, which provide a cumulative record of dietary intake over several days. The complex 3D architecture and high spider density of the subtropical canopy likely facilitate frequent encounters between guilds, a pattern supported by our heatmap showing multiple families preying on Nephilidae spiderlings during their seasonal dispersal. Furthermore, our high starvation rate strongly argues against systemic contamination; if contamination were present, it would be impossible to observe such a high proportion of empty guts. Crucially, a sensitivity analysis excluding all Araneae prey confirmed that the core ecological patterns of niche partitioning and the functional hierarchy of generality remained remarkably consistent. This demonstrates that the high incidence of intraguild predation reflects the intense trophic connectivity of this forest canopy and does not bias our broader conclusions regarding spider functional roles (Wise et al. 2023). Intraguild predation is a common phenomenon among spiders, as it serves as an effective strategy for acquiring high-quality food resources (Michalko et al. 2021b). Given the limitation of metabarcoding in differentiating closely related species, the actual incidence of intraguild predation was likely underestimated in our study (and might also involve cannibalism within the target species; Wise et al. 2023). This finding aligns with previous research from the forest floor, suggesting higher intraguild predation rates among spiders in summer compared to other seasons, a pattern potentially linked to life history traits such as breeding cycles (Wise et al. 2023). While active hunters are often expected to exhibit higher intraguild predation due to frequent encounters, our findings reveal that web-building spiders also engage in substantial levels of intraguild predation (Wise 2006; Wise et al. 2023). Web-building spiders also compete intensely for suitable web-building space and attachment points. Specifically, larger spiders often exhibit competitive exclusion toward smaller individuals, a phenomenon that becomes particularly pronounced during periods of scarce food resources (Yip et al. 2017). The high starvation rate observed may have contributed to the high intraguild predation, as starving individuals can become more aggressive, leading them to attack conspecifics despite the inherent risk of being preyed upon (Petersen et al. 2010; Scharf 2016). While the underlying drivers of the high intraguild predation levels in this spider community warrant further investigation, its prevalence underscores the dual ecological roles of these predators: they not only regulate communities at lower trophic levels but also modulate the overall strength of this top-down effect, ultimately influencing ecological functions, such as biological pest control (Michalko et al. 2019; Uiterwaal et al. 2023).

Our results revealed distinct contrasts in the feeding characteristics of hunting and web-building spiders. Hunting spiders had a lower starvation rate and a more diverse diet, characterized by greater dietary breadth and overlap. In contrast, web-building spiders showed a significantly higher starvation rate and lower prey richness, resulting in a narrower diet. This pattern aligns with our hypothesis (a). Crucially, there was no significant deviation from the null model for the web-building spider network.This suggests that web-builders lack significant prey preferences, with capture rates largely driven by prey abundance and random encounter probabilities. These factors, in turn, are influenced by environmental diversity and complexity (Diehl et al. 2013; Mader et al. 2016). Conversely, hunting spiders showed significantly lower niche overlap and generality than expected by chance, indicating a more selective and segregated foraging strategy enabled by their active hunting behavior. These contrasting findings suggest that spiders with different hunting modes employ distinct survival strategies. Web-building spiders, as “sit-and-wait” predators, invest significant energy upfront in web construction, and their subsequent prey capture success is expected to be physically constrained by web characteristics (e.g., web size, mesh size, etc., Ludwig et al. 2018). Our null model results suggest that web-builders may function as non-selective foragers highly dependent on local prey density. This passive strategy, while potentially capturing multiple prey, exposes them to the risk of prolonged periods without successful foraging (Wimp et al. 2013). In contrast, hunting spiders are active foragers that continuously expend energy to search for prey (Willemart and Lacava 2017). This proactive approach appears to provide more consistent and frequent feeding opportunities, thereby reducing their starvation rate and diversifying their diet. This strategic divergence is further corroborated by established physiological differences: web-building spiders typically have lower metabolic rates and greater starvation tolerance than hunting spiders (Nagel et al. 2025), adaptations that are well-suited to their respective foraging strategies. Furthermore, the high mobility of hunting spiders facilitates frequent encounters with diverse prey, driving the high dietary breadth and overlap, which concomitantly increases potential competition among active hunters (Baatrup et al. 2018). Unlike web-builders constrained by their rather static webs, hunting spiders appear to utilize their mobility to mitigate competition by partitioning resources, which is suggested by the lower than expected (based on null models) dietary overlap and niche differentiation. The distinct prey preferences of the two foraging guilds highlight their different ecological functions (Chen et al. 2025; Nyffeler 1999). This underscores the significant role of varied predation patterns in regulating the top-down effects exerted by spiders and emphasizes the importance of integrating hunting strategies into future studies of predator-prey interaction networks.

Among the dominant spider families, we found that beyond the hunting mode, intrinsic functional characteristics (e.g., web architecture) significantly shape their feeding preferences and interaction structures, potentially altering the pathways of top-down effects on lower trophic levels. Specifically, Araneidae exhibited a higher starvation rate compared to Theridiidae, but simultaneously maintained a broader diet width and lower dietary similarity, aligning with our hypothesis (b). This pattern is likely attributed to the design of Araneidae orb-webs, specifically their large overall size and mesh dimension (Blamires et al. 2017). Large mesh sizes may selectively filter out abundant small prey, biasing capture toward larger but less frequently encountered prey items. This reliance on sporadic high-quality prey likely contributes to their observed high starvation tolerance (Nagel et al. 2025). In contrast, Theridiidae construct small yet complex three-dimensional webs. This web structure likely limits the breadth of captured prey (Ludwig et al. 2018), yet it also promotes higher spider density (due to spatial proximity) and potentially increased predation efficiency. Consequently, this leads to fewer starved individuals and a more homogeneous diet across the population (Japyassú and Caires 2008). Among hunting spiders, both Oxyopidae and Thomisidae emerged as highly effective predators, evidenced by the majority of sampled individuals containing detectable prey DNA. This observation suggests that both feeding strategies — active searching (Oxyopidae) and sit-and-wait ambushing (Thomisidae) — are efficient means of prey capture, only partially aligning with our hypothesis (c). The active searching strategy employed by Oxyopidae has higher probability of encountering a wider array of prey items, potentially contributing to their increased dietary breadth (Memah et al. 2018; Tahir and Butt 2009). Thomisidae, however, typically position themselves in specific microhabitats within the tree canopy (e.g. leaves, flowers and branches etc., Evans 1997; Jiménez-Valverde and Lobo 2007), which may result in more homogeneous dietary patterns. Unexpectedly, we observed a high starvation rate in Salticidae, which appears counterintuitive given exceptionally acute vision, a key function for active hunting (Cerveira et al. 2021; Morehouse 2020). Oxyopidae and Salticidae showed significantly lower niche overlap and generality than expected by chance, which indicates that active foragers exert strong selective pressure on prey and exhibit clear niche partitioning, likely to minimize interspecific competition. Specifically for Oxyopidae, the observed pattern of high generalism coupled with significant niche differentiation may be largely driven by the dominant presence of a single, highly abundant species within the study site.

Comparison of dietary similarity among different spider guilds revealed a relatively large overlap of prey at high taxonomic levels and within functional groups. This observation is expected, as spiders are well-known generalist predators. Furthermore, the strongly increasing prey species accumulation curves for most spider groups indicate that the diet of individual spiders varies substantially, suggesting that species-rich spider communities collectively cover an extremely broad range of potential prey organisms. This broad dietary scope aligns with the overall relatively low vulnerability values observed, which indicate that any given prey species faces a comparatively low probability of being attacked by a single, specific spider species. Consequently, we propose that the full potential of spiders as top-down regulators in food webs depends not solely on the functional diversity within and among guilds or families, but critically on the sheer abundance (or population density) and species richness of the spider community.

Although high-throughput sequencing provides a powerful tool for analyzing spider-prey interaction networks (Cuff et al. 2022; Evans et al. 2016), the current approach has several limitations that require careful consideration. First, our use of the proportion of individuals with no detectable prey as a proxy for the starvation rate of spiders must be interpreted with caution. Prey DNA detection from the digestion system depends on numerous factors, including prey biomass, digestion duration (Macías-Hernández et al. 2018), predator metabolic rates and PCR primer specificity (Cuff et al. 2022). Thus, DNA from small or rapidly-digested prey may be undetected due to PCR amplification, potentially inflating starvation rate estimates. Despite these methodological limitations, the high frequency of spiders lacking detectable prey DNA still indicates substantial food stress within the community. One shortcoming of the molecular methodology employed here is its failure to account for intraspecific cannibalism. To better explore these intraspecific interactions in the future, it will be necessary to adopt multi-method approaches or higher-resolution molecular assays that can differentiate between individuals of the same species. Second, the sampling of this study was limited to a single snapshot in time and lacked assessments of environmental prey availability. A complete characterization of spider diet ecology would require more comprehensive sampling across different seasons and vegetation strata. Furthermore, incorporating local prey resource is essential to disentangle the underlying mechanisms driving spider foraging patterns and preferences. Finally, using a single primer pair inherently introduces taxonomic amplification bias. To achieve a more accurate identification of diet composition, a multi-primer approach should be adopted in further diet analysis with metabarcoding (Cuff et al. 2023; Ficetola and Taberlet 2023).

Overall, our study provides a detailed characterization of the arboreal spider-prey interaction network within a subtropical forest, underscoring the prevalence and structural importance of intraguild predation in this community. Critically, our findings demonstrate that the hunting mode of spiders is a key determinant of top-down effects, shaping both direct predation on lower trophic levels and the intensity of intraguild predation. Altogether, these findings call for careful consideration of the spider community structure when using spiders for biological control in order to avoid inhibitory trophic cascades arising from high intraguild predation. Ultimately, these insights into guild-specific predation pressures and network properties underscore the critical importance of resolving fine-scale trophic interactions to fully understand food web structure and the multifaceted functional roles of spiders in ecosystems.

## Supplementary Information

Below is the link to the electronic supplementary material.

Supplementary Material 1

## Data Availability

Data is available on the Science Data Bank https://doi.org/10.57760/sciencedb.45397 and BEF-China project database https://data.botanik.uni-halle.de/bef-china/datasets/771.
