# Supplementary material for: Metabarcoding reveals the dietary diversity and food web structure of spider functional guilds in a highly diverse subtropical forest
Source: Oecologia. 2026 Aug 7;208(9):108. doi: 10.1007/s00442-026-05956-9 (PMC13451308; doi:10.1007/s00442-026-05956-9)
Supplement: Supplementary file 1 [file 442_2026_5956_MOESM1_ESM.docx]

**Metabarcoding reveals the dietary diversity and food web structure of spider functional guilds in a highly diverse subtropical forest**

**Supplementary Information**

**Chen et al.**

**Fig S1.** Histogram depicting the distribution of pairwise genetic distances among host spider DNA sequences obtained through metabarcoding, calculated using the K80 substitution model.

**Figure S2** Nonmetric multidimensional scaling (NMDS) analysis showing the prey composition of (a) spiders in two hunting modes (web-building and hunting), (b) spiders of five dominant families (Araneidae, Theridiidae, Oxyopidae, Salticidae, Thomisidae). Ellipses represent the standard deviation around the centroids of spiders in different hunting modes and dominant families.

**Figure S3** Mean relative prey richness across spider guilds and families based on a standardized bootstrap resampling procedure (n = 100 individuals per group, 999 iterations).

**Figure S4** Heatmap of family-level intraguild predation (IGP) among spiders. The color intensity (white to red) represents the number of individual spiders in which a specific predator-prey interactions was detected with DNA barcoding.

**Figure S5** Sensitivity analysis using the insect-only dataset: prey richness and network structure. (a) Mean relative richness of insect prey detected across all spiders, the two hunting guilds, and five dominant families. (b) Bipartite predator-prey interaction networks resolved at the MOTU level, where boxes represent individual predator/prey MOTUs and linking lines indicate detected feeding interactions. The left column represents predator spider MOTUs, and the right column represents insect prey MOTUs.

**Figure S6** Rarefaction and extrapolation sampling curves of prey richness based on the insect-only dataset. The curves illustrate the accumulation of prey species richness as a function of the number of spider species sampled for (a) the total dataset and the two hunting guilds, and (b) the five dominant spider families. Shaded areas represent 95% confidence intervals.

**Figure S7** Non-metric multidimensional scaling (NMDS) ordination of insect prey composition. NMDS plots illustrate dietary differentiation based on the insect-only dataset for (a) the two primary hunting guilds (web-building vs. active hunting) and (b) the five dominant spider families (Araneidae, Theridiidae, Oxyopidae, Salticidae, and Thomisidae). Ellipses represent the standard deviation (SD) around the centroids for each group.

**Figure S8** Functional group composition of insect prey across spider groups. Proportions of various functional groups (e.g., herbivores, carnivores, and decomposers) within the insect prey spectrum captured by all spiders, the two hunting guilds, and the five dominant families.

**Table S1** Comparison of observed network metrics with null model expectations across different spider guilds. The network metrics include niche overlap, generality and vulnerability for all spiders, two feeding guilds and five dominant families. Null models were generated using the Patefield algorithm (N = 10000). *P* value indicates the significance of the deviation of observed values from random expectations.

|  |  | Niche overlap | Generality | Vulnerability |
| --- | --- | --- | --- | --- |
| All | Observed Value | 0.12 | 19.98 | 6.22 |
|  | Null Mean | 0.15 | 31.03 | 8.66 |
|  | *P* Value | **0.007** | **0.000** | **0.000** |
| Web | Observed Value | 0.08 | 19.14 | 3.93 |
|  | Null Mean | 0.07 | 20.18 | 4.00 |
|  | *P* Value | 0.118 | 0.114 | 0.398 |
| Hunt | Observed Value | 0.16 | 20.39 | 4.21 |
|  | Null Mean | 0.21 | 29.43 | 5.52 |
|  | *P* Value | **0.049** | **0.000** | **0.000** |
| Araneidae | Observed Value | 0.04 | 25.43 | 1.88 |
|  | Null Mean | 0.04 | 29.47 | 2.18 |
|  | *P* Value | 0.369 | **0.000** | **0.001** |
| Theridiidae | Observed Value | 0.12 | 7.10 | 2.58 |
|  | Null Mean | 0.14 | 8.04 | 2.83 |
|  | *P* Value | 0.217 | **0.039** | 0.152 |
| Oxyopidae | Observed Value | 0.06 | 31.75 | 1.23 |
|  | Null Mean | 0.18 | 34.45 | 1.34 |
|  | *P* Value | **0.003** | **0.017** | **0.004** |
| Thomisidae | Observed Value | 0.35 | 16.43 | 2.32 |
|  | Null Mean | 0.29 | 21.04 | 2.78 |
|  | *P* Value | 0.157 | **0.000** | **0.000** |
| Salticidae | Observed Value | 0.13 | 12.84 | 2.60 |
|  | Null Mean | 0.23 | 15.17 | 3.61 |
|  | *P* Value | **0.001** | **0.000** | **0.000** |

**Table S2** Dietary metrics and network indices derived from the insect-only sensitivity analysis. Summary of observed prey richness, starvation ratios (empty gut frequencies), and network indices for all spider groups and dominant families. Asterisks (*) indicate observed values that were significantly lower than expectations derived from null model analyses.

|  | Prey richness | Starvation ratio | Niche overlap | Generality | Vulnerability |
| --- | --- | --- | --- | --- | --- |
| All | 271 | 81.77 | 0.02 | 26.84 | 2.28 |
| Web | 131 | 86.92 | 0.02 | 21.38 | 1.72 |
| Hunt | 177 | 72.09 | 0.03 | 30.70 | 1.69 |
| Araneidae | 112 | 88.11 | 0.01 | 25.12 | 1.50 |
| Theridiidae | 16 | 77.08 | 0.01 | 3.40 | 1.15 |
| Oxyopidae | 70 | 55.08 | 0.03 | 47.26 | 1.11 |
| Thomisidae | 81 | 60.26 | 0.01 | 29.28 | 1.11 |
| Salticidae | 39 | 85.53 | 0.13 | 7.86 | 1.50 |

**Table S3** Statistical comparison of observed network metrics with null model expectations for the insect-only dataset. The table shows niche overlap, generality, and vulnerability indices for all spider guilds and families. Null models were generated using the Patefield algorithm (*N* = 10,000 iterations). *P*-values indicate the significance of the deviation of observed network structures from random-encounter expectations.

|  |  | Niche overlap | Generality | Vulnerability |
| --- | --- | --- | --- | --- |
| All | Observed Value | 0.02 | 26.84 | 2.28 |
|  | Null Mean | 0.02 | 30.65 | 2.72 |
|  | *P* Value | 0.258 | **0.000** | **0.001** |
| Web | Observed Value | 0.02 | 21.38 | 1.72 |
|  | Null Mean | 0.01 | 22.49 | 1.79 |
|  | *P* Value | 0.173 | 0.093 | 0.210 |
| Hunt | Observed Value | 0.03 | 30.70 | 1.69 |
|  | Null Mean | 0.05 | 33.93 | 2.08 |
|  | *P* Value | **0.020** | **0.012** | **0.000** |
| Araneidae | Observed Value | 0.01 | 25.12 | 1.50 |
|  | Null Mean | 0.02 | 26.58 | 1.57 |
|  | *P* Value | **0.000** | **0.000** | **0.101** |
| Theridiidae | Observed Value | 0.01 | 3.40 | 1.15 |
|  | Null Mean | 0.03 | 3.692 | 1.25 |
|  | *P* Value | 0.264 | 0.32 | 0.413 |
| Oxyopidae | Observed Value | 0.03 | 47.26 | 1.11 |
|  | Null Mean | 0.04 | 48.31 | 1.13 |
|  | *P* Value | 0.408 | 0.283 | 0.336 |
| Thomisidae | Observed Value | 0.01 | 29.28 | 1.11 |
|  | Null Mean | 0.06 | 31.70 | 1.44 |
|  | *P* Value | **0.000** | **0.100** | **0.000** |
| Salticidae | Observed Value | 0.13 | 7.86 | 1.50 |
|  | Null Mean | 0.23 | 7.82 | 1.81 |
|  | *P* Value | **0.013** | 0.494 | **0.017** |
